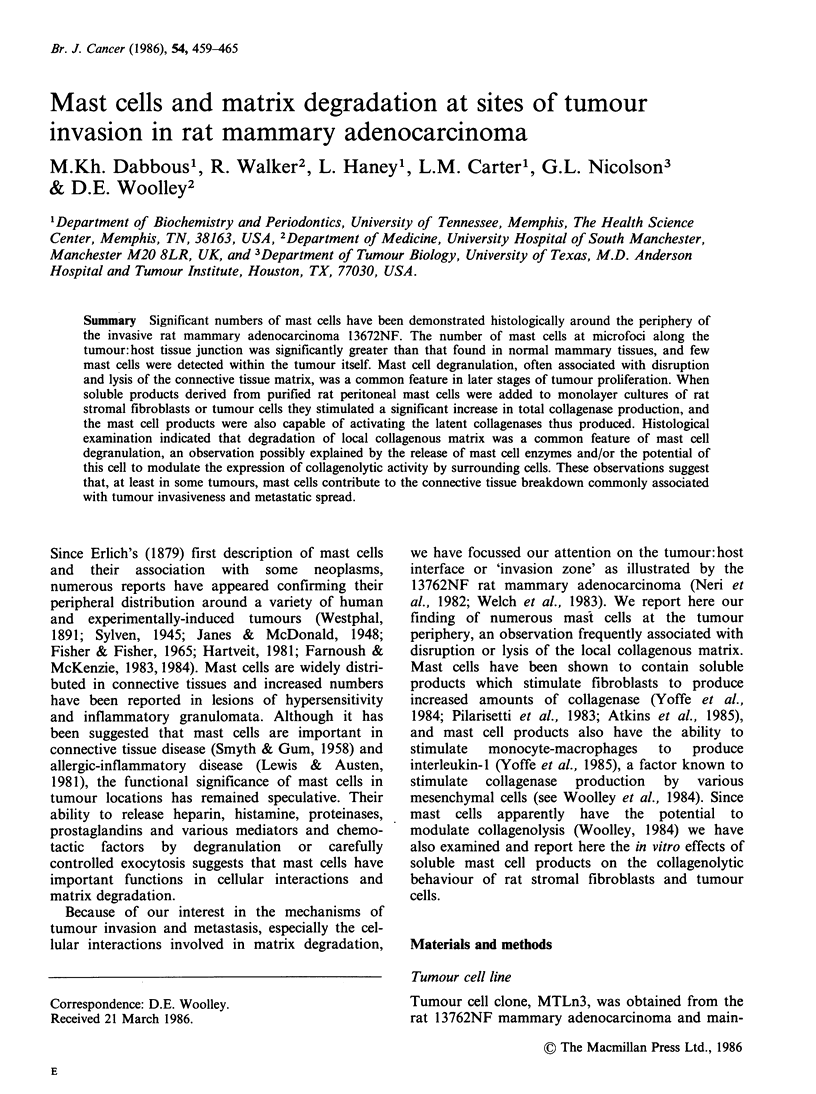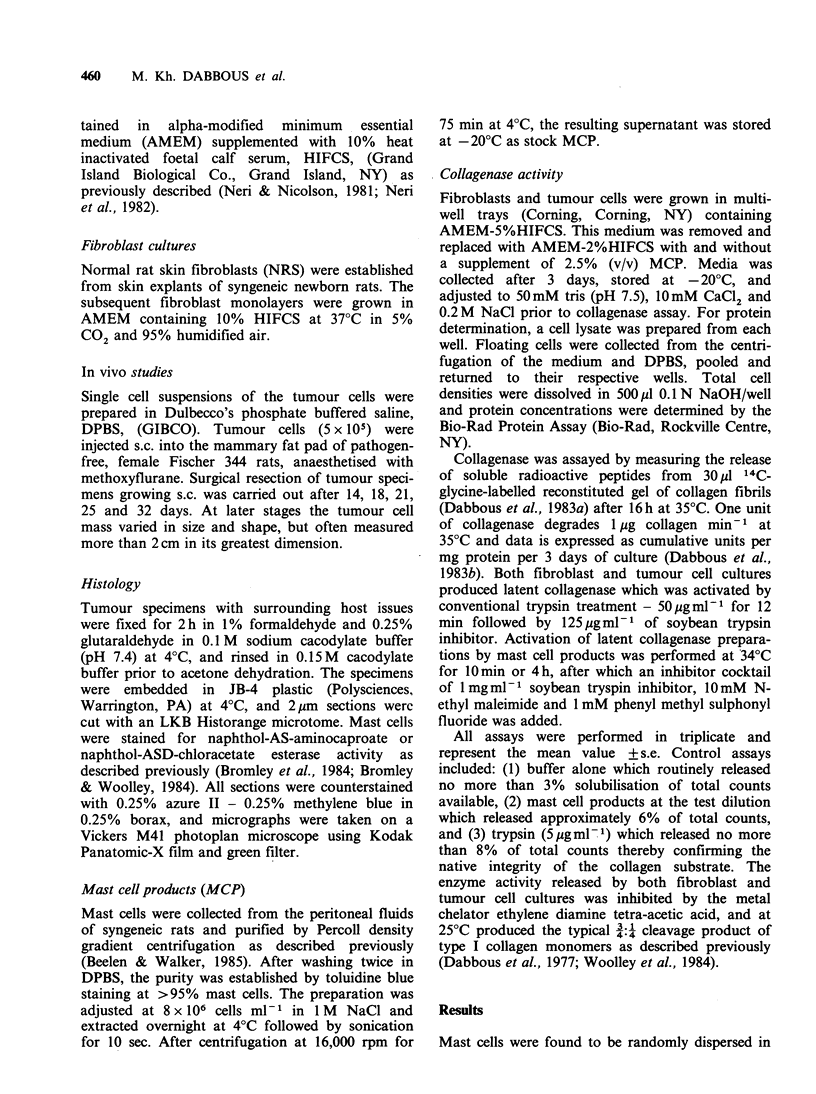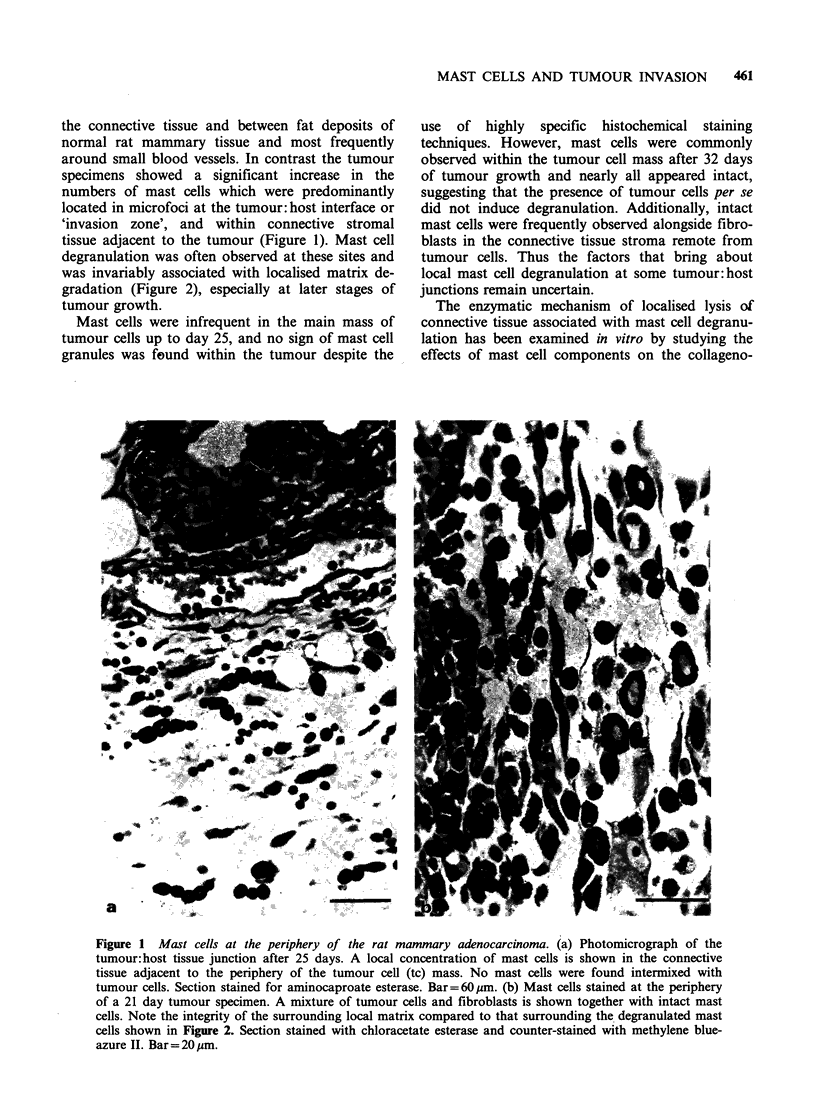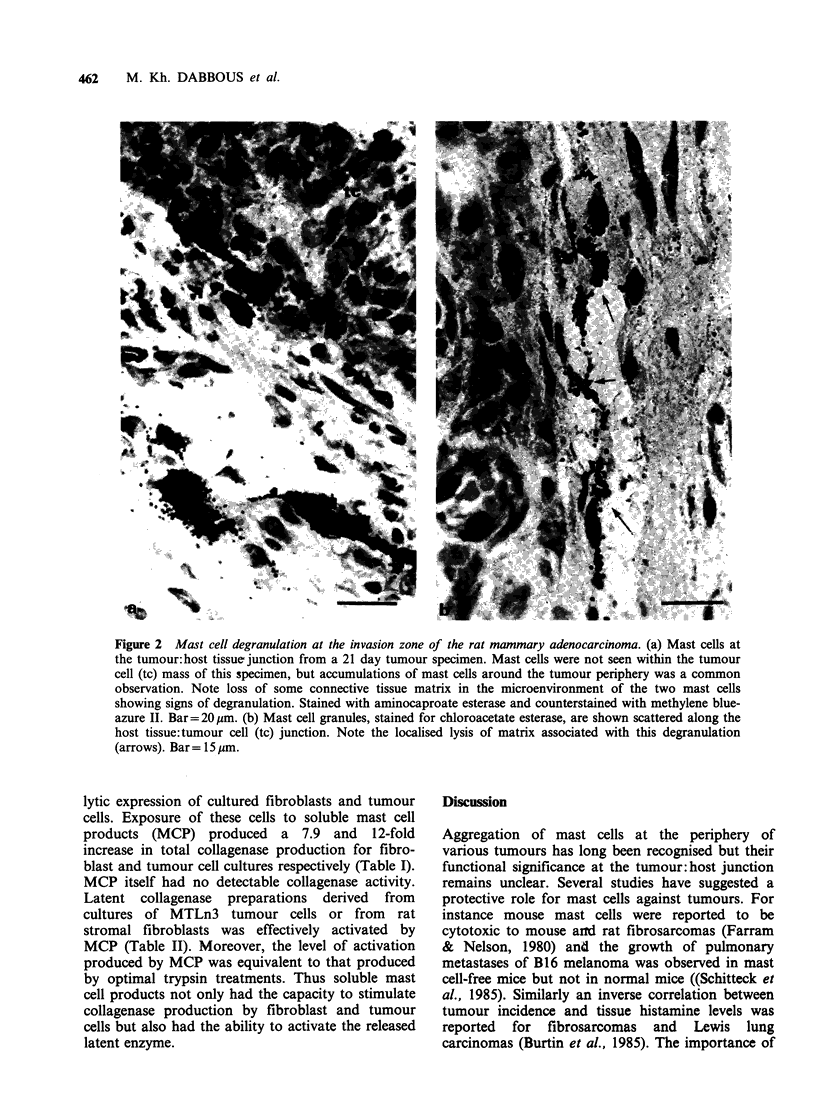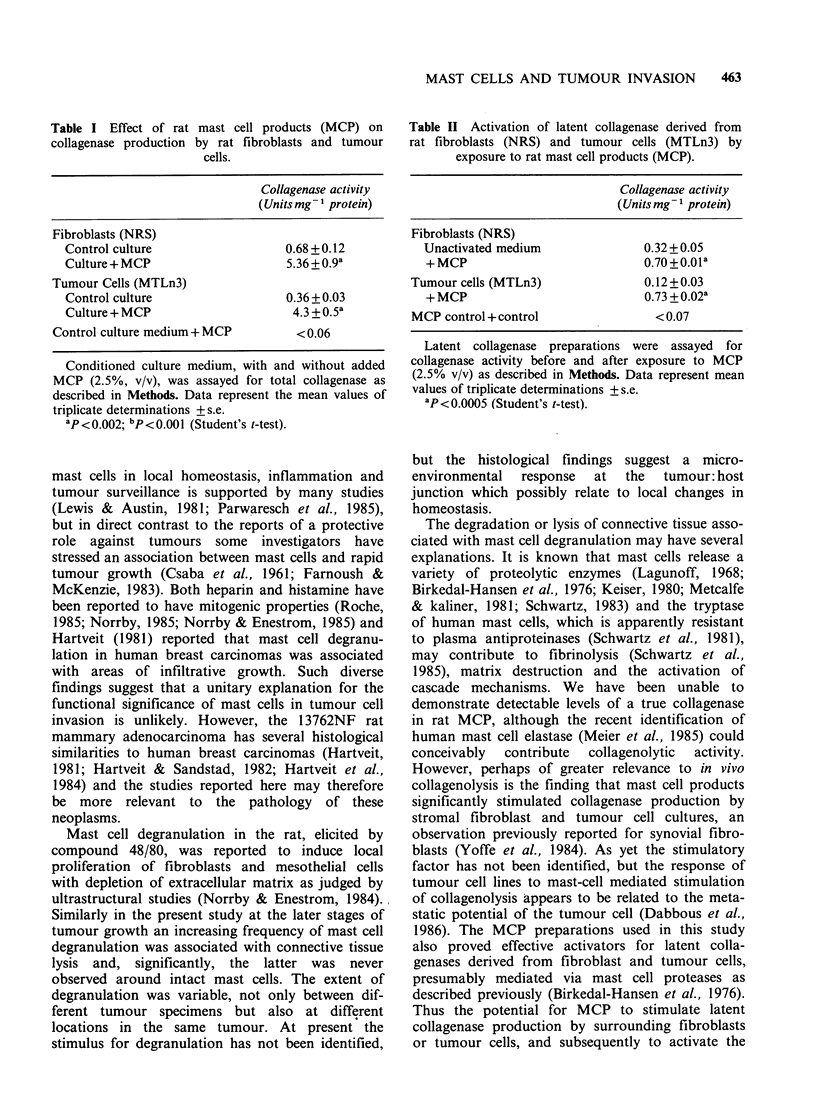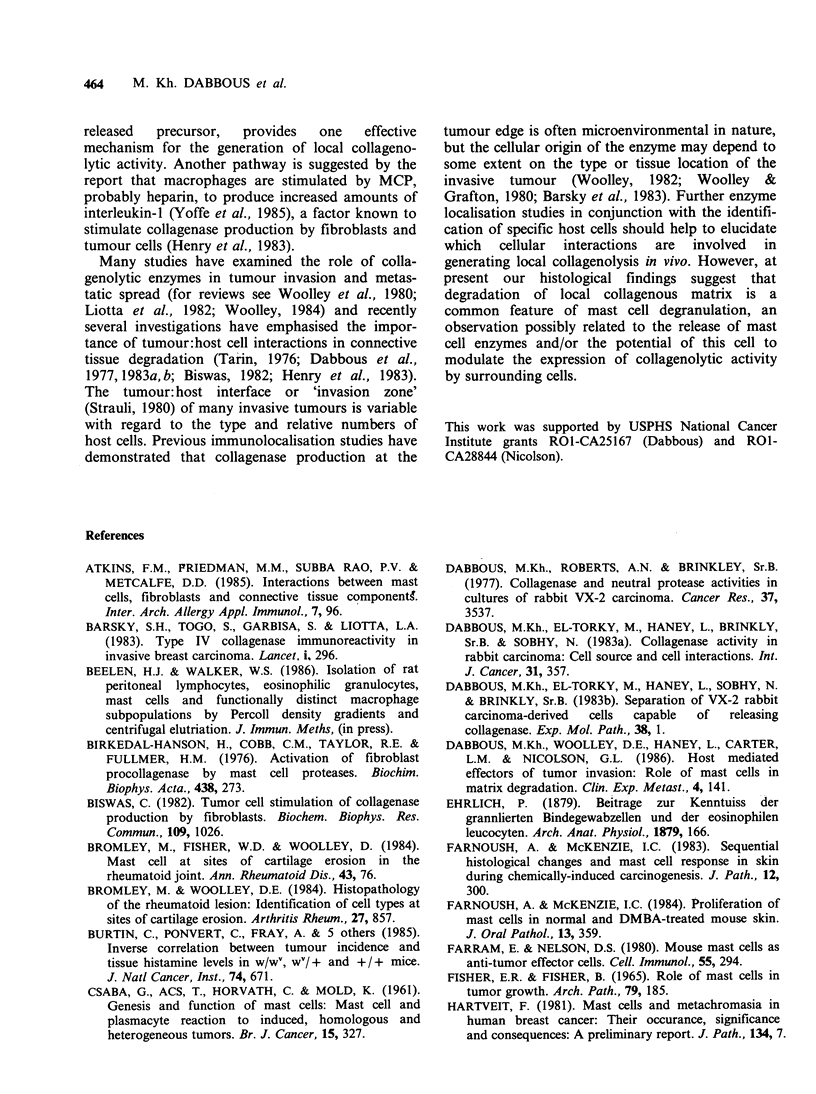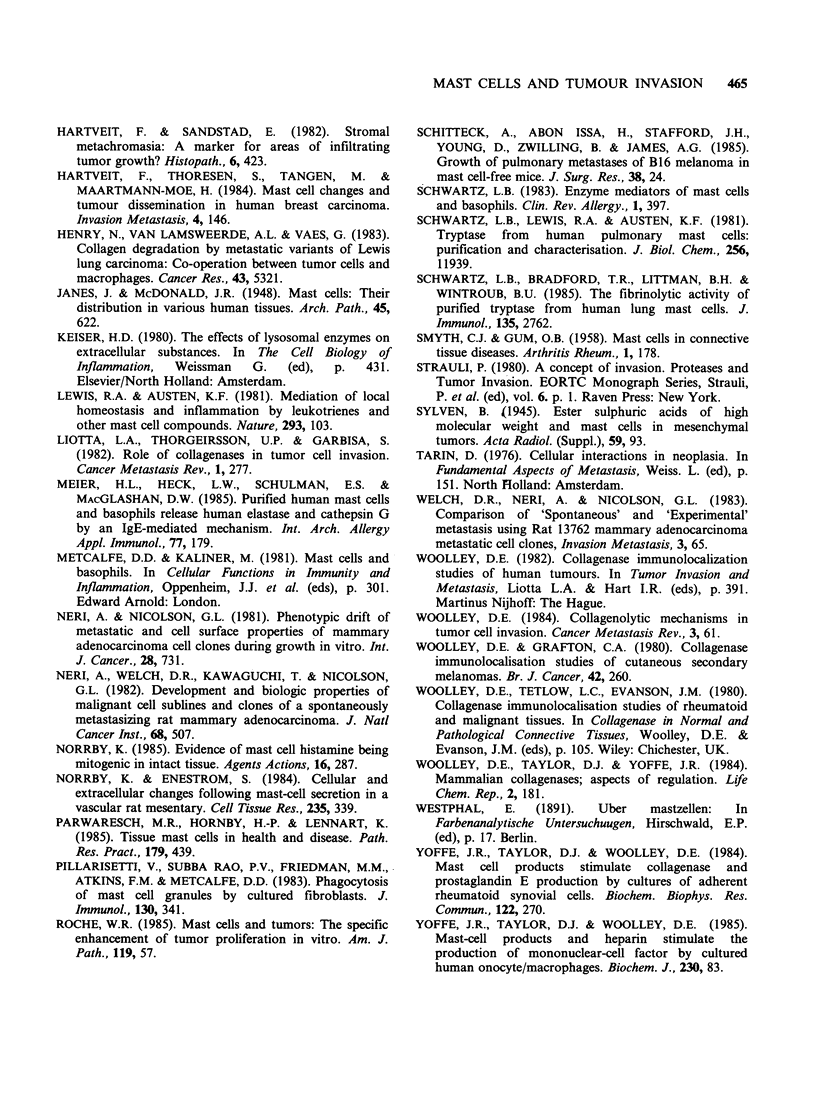# Mast cells and matrix degradation at sites of tumour invasion in rat mammary adenocarcinoma.

**DOI:** 10.1038/bjc.1986.198

**Published:** 1986-09

**Authors:** M. K. Dabbous, R. Walker, L. Haney, L. M. Carter, G. L. Nicolson, D. E. Woolley

## Abstract

**Images:**


Br. J. Cancer (1986), 54, 459–465

### Mast cells and matrix degradation at sites of tumour invasion in rat mammary adenocarcinoma

M.Kh. Dabbous[1], R. Walker[2], L. Haney[1], L.M. Carter[1], G.L. Nicolson[3] & D.E. Woolley[2]

[1]*Department of Biochemistry and Periodontics, University of Tennessee, Memphis, The Health Science Center, Memphis, TN, 38163, USA,* [2]*Department of Medicine, University Hospital of South Manchester, Manchester M20 8LR, UK, and* [3]*Department of Tumour Biology, University of Texas, M.D. Anderson Hospital and Tumour Institute, Houston, TX, 77030, USA.*

**Summary** Significant numbers of mast cells have been demonstrated histologically around the periphery of the invasive rat mammary adenocarcinoma 13672NF. The number of mast cells at microfoci along the tumour:host tissue junction was significantly greater than that found in normal mammary tissues, and few mast cells were detected within the tumour itself. Mast cell degranulation, often associated with disruption and lysis of the connective tissue matrix, was a common feature in later stages of tumour proliferation. When soluble products derived from purified rat peritoneal mast cells were added to monolayer cultures of rat stromal fibroblasts or tumour cells they stimulated a significant increase in total collagenase production, and the mast cell products were also capable of activating the latent collagenases thus produced. Histological examination indicated that degradation of local collagenous matrix was a common feature of mast cell degranulation, an observation possibly explained by the release of mast cell enzymes and/or the potential of this cell to modulate the expression of collagenolytic activity by surrounding cells. These observations suggest that, at least in some tumours, mast cells contribute to the connective tissue breakdown commonly associated with tumour invasiveness and metastatic spread.

Since Erlich's (1879) first description of mast cells and their association with some neoplasms, numerous reports have appeared confirming their peripheral distribution around a variety of human and experimentally-induced tumours (Westphal, 1891; Sylven, 1945; Janes & McDonald, 1948; Fisher & Fisher, 1965; Hartveit, 1981; Farnoush & McKenzie, 1983, 1984). Mast cells are widely distributed in connective tissues and increased numbers have been reported in lesions of hypersensitivity and inflammatory granulomata. Although it has been suggested that mast cells are important in connective tissue disease (Smyth & Gum, 1958) and allergic-inflammatory disease (Lewis & Austen, 1981), the functional significance of mast cells in tumour locations has remained speculative. Their ability to release heparin, histamine, proteinases, prostaglandins and various mediators and chemotactic factors by degranulation or carefully controlled exocytosis suggests that mast cells have important functions in cellular interactions and matrix degradation.

Because of our interest in the mechanisms of tumour invasion and metastasis, especially the cellular interactions involved in matrix degradation, we have focussed our attention on the tumour:host interface or 'invasion zone' as illustrated by the 13762NF rat mammary adenocarcinoma (Neri *et al.*, 1982; Welch *et al.*, 1983). We report here our finding of numerous mast cells at the tumour periphery, an observation frequently associated with disruption or lysis of the local collagenous matrix. Mast cells have been shown to contain soluble products which stimulate fibroblasts to produce increased amounts of collagenase (Yoffe *et al.*, 1984; Pilarisetti *et al.*, 1983; Atkins *et al.*, 1985), and mast cell products also have the ability to stimulate monocyte-macrophages to produce interleukin-1 (Yoffe *et al.*, 1985), a factor known to stimulate collagenase production by various mesenchymal cells (see Woolley *et al.*, 1984). Since mast cells apparently have the potential to modulate collagenolysis (Woolley, 1984) we have also examined and report here the *in vitro* effects of soluble mast cell products on the collagenolytic behaviour of rat stromal fibroblasts and tumour cells.

### Materials and methods

### *Tumour cell line*

Tumour cell clone, MTLn3, was obtained from the rat 13762NF mammary adenocarcinoma and main-

Correspondence: D.E. Woolley.
Received 21 March 1986.

© The Macmillan Press Ltd., 1986

tained in alpha-modified minimum essential medium (AMEM) supplemented with 10% heat inactivated foetal calf serum, HIFCS, (Grand Island Biological Co., Grand Island, NY) as previously described (Neri & Nicolson, 1981; Neri *et al.*, 1982).

*Fibroblast cultures*

Normal rat skin fibroblasts (NRS) were established from skin explants of syngeneic newborn rats. The subsequent fibroblast monolayers were grown in AMEM containing 10% HIFCS at 37°C in 5% $CO_2$ and 95% humidified air.

In vivo *studies*

Single cell suspensions of the tumour cells were prepared in Dulbecco's phosphate buffered saline, DPBS, (GIBCO). Tumour cells ($5 \times 10^5$) were injected s.c. into the mammary fat pad of pathogen-free, female Fischer 344 rats, anaesthetised with methoxyflurane. Surgical resection of tumour specimens growing s.c. was carried out after 14, 18, 21, 25 and 32 days. At later stages the tumour cell mass varied in size and shape, but often measured more than 2 cm in its greatest dimension.

*Histology*

Tumour specimens with surrounding host issues were fixed for 2 h in 1% formaldehyde and 0.25% glutaraldehyde in 0.1 M sodium cacodylate buffer (pH 7.4) at 4°C, and rinsed in 0.15 M cacodylate buffer prior to acetone dehydration. The specimens were embedded in JB-4 plastic (Polysciences, Warrington, PA) at 4°C, and 2 $\mu$m sections were cut with an LKB Historange microtome. Mast cells were stained for naphthol-AS-aminocaproate or naphthol-ASD-chloracetate esterase activity as described previously (Bromley *et al.*, 1984; Bromley & Woolley, 1984). All sections were counterstained with 0.25% azure II – 0.25% methylene blue in 0.25% borax, and micrographs were taken on a Vickers M41 photoplan microscope using Kodak Panatomic-X film and green filter.

*Mast cell products (MCP)*

Mast cells were collected from the peritoneal fluids of syngeneic rats and purified by Percoll density gradient centrifugation as described previously (Beelen & Walker, 1985). After washing twice in DPBS, the purity was established by toluidine blue staining at >95% mast cells. The preparation was adjusted at $8 \times 10^6$ cells $ml^{-1}$ in 1 M NaCl and extracted overnight at 4°C followed by sonication for 10 sec. After centrifugation at 16,000 rpm for 75 min at 4°C, the resulting supernatant was stored at −20°C as stock MCP.

*Collagenase activity*

Fibroblasts and tumour cells were grown in multi-well trays (Corning, Corning, NY) containing AMEM-5%HIFCS. This medium was removed and replaced with AMEM-2%HIFCS with and without a supplement of 2.5% (v/v) MCP. Media was collected after 3 days, stored at −20°C, and adjusted to 50 mM tris (pH 7.5), 10 mM $CaCl_2$ and 0.2 M NaCl prior to collagenase assay. For protein determination, a cell lysate was prepared from each well. Floating cells were collected from the centrifugation of the medium and DPBS, pooled and returned to their respective wells. Total cell densities were dissolved in 500 $\mu$l 0.1 N NaOH/well and protein concentrations were determined by the Bio-Rad Protein Assay (Bio-Rad, Rockville Centre, NY).

Collagenase was assayed by measuring the release of soluble radioactive peptides from 30 $\mu$l $^{14}C$-glycine-labelled reconstituted gel of collagen fibrils (Dabbous *et al.*, 1983*a*) after 16 h at 35°C. One unit of collagenase degrades 1 $\mu$g collagen $min^{-1}$ at 35°C and data is expressed as cumulative units per mg protein per 3 days of culture (Dabbous *et al.*, 1983*b*). Both fibroblast and tumour cell cultures produced latent collagenase which was activated by conventional trypsin treatment – 50 $\mu g\,ml^{-1}$ for 12 min followed by 125 $\mu g\,ml^{-1}$ of soybean trypsin inhibitor. Activation of latent collagenase preparations by mast cell products was performed at 34°C for 10 min or 4 h, after which an inhibitor cocktail of 1 $mg\,ml^{-1}$ soybean tryspin inhibitor, 10 mM N-ethyl maleimide and 1 mM phenyl methyl sulphonyl fluoride was added.

All assays were performed in triplicate and represent the mean value ±s.e. Control assays included: (1) buffer alone which routinely released no more than 3% solubilisation of total counts available, (2) mast cell products at the test dilution which released approximately 6% of total counts, and (3) trypsin (5 $\mu g\,ml^{-1}$) which released no more than 8% of total counts thereby confirming the native integrity of the collagen substrate. The enzyme activity released by both fibroblast and tumour cell cultures was inhibited by the metal chelator ethylene diamine tetra-acetic acid, and at 25°C produced the typical $\frac{3}{4}$:$\frac{1}{4}$ cleavage product of type I collagen monomers as described previously (Dabbous *et al.*, 1977; Woolley *et al.*, 1984).

### Results

Mast cells were found to be randomly dispersed in the connective tissue and between fat deposits of normal rat mammary tissue and most frequently around small blood vessels. In contrast the tumour specimens showed a significant increase in the numbers of mast cells which were predominantly located in microfoci at the tumour:host interface or 'invasion zone', and within connective stromal tissue adjacent to the tumour (Figure 1). Mast cell degranulation was often observed at these sites and was invariably associated with localised matrix degradation (Figure 2), especially at later stages of tumour growth.

Mast cells were infrequent in the main mass of tumour cells up to day 25, and no sign of mast cell granules was found within the tumour despite the use of highly specific histochemical staining techniques. However, mast cells were commonly observed within the tumour cell mass after 32 days of tumour growth and nearly all appeared intact, suggesting that the presence of tumour cells *per se* did not induce degranulation. Additionally, intact mast cells were frequently observed alongside fibroblasts in the connective tissue stroma remote from tumour cells. Thus the factors that bring about local mast cell degranulation at some tumour:host junctions remain uncertain.

The enzymatic mechanism of localised lysis of connective tissue associated with mast cell degranulation has been examined *in vitro* by studying the effects of mast cell components on the collageno-

**Figure 1** *Mast cells at the periphery of the rat mammary adenocarcinoma.* (a) Photomicrograph of the tumour:host tissue junction after 25 days. A local concentration of mast cells is shown in the connective tissue adjacent to the periphery of the tumour cell (tc) mass. No mast cells were found intermixed with tumour cells. Section stained for aminocaproate esterase. Bar = 60 $\mu$m. (b) Mast cells stained at the periphery of a 21 day tumour specimen. A mixture of tumour cells and fibroblasts is shown together with intact mast cells. Note the integrity of the surrounding local matrix compared to that surrounding the degranulated mast cells shown in **Figure 2.** Section stained with chloracetate esterase and counter-stained with methylene blue-azure II. Bar = 20 $\mu$m.

**Figure 2** *Mast cell degranulation at the invasion zone of the rat mammary adenocarcinoma.* (a) Mast cells at the tumour:host tissue junction from a 21 day tumour specimen. Mast cells were not seen within the tumour cell (tc) mass of this specimen, but accumulations of mast cells around the tumour periphery was a common observation. Note loss of some connective tissue matrix in the microenvironment of the two mast cells showing signs of degranulation. Stained with aminocaproate esterase and counterstained with methylene blue-azure II. Bar = 20 μm. (b) Mast cell granules, stained for chloroacetate esterase, are shown scattered along the host tissue:tumour cell (tc) junction. Note the localised lysis of matrix associated with this degranulation (arrows). Bar = 15 μm.

lytic expression of cultured fibroblasts and tumour cells. Exposure of these cells to soluble mast cell products (MCP) produced a 7.9 and 12-fold increase in total collagenase production for fibroblast and tumour cell cultures respectively (Table I). MCP itself had no detectable collagenase activity. Latent collagenase preparations derived from cultures of MTLn3 tumour cells or from rat stromal fibroblasts was effectively activated by MCP (Table II). Moreover, the level of activation produced by MCP was equivalent to that produced by optimal trypsin treatments. Thus soluble mast cell products not only had the capacity to stimulate collagenase production by fibroblast and tumour cells but also had the ability to activate the released latent enzyme.

### Discussion

Aggregation of mast cells at the periphery of various tumours has long been recognised but their functional significance at the tumour:host junction remains unclear. Several studies have suggested a protective role for mast cells against tumours. For instance mouse mast cells were reported to be cytotoxic to mouse and rat fibrosarcomas (Farram & Nelson, 1980) and the growth of pulmonary metastases of B16 melanoma was observed in mast cell-free mice but not in normal mice ((Schitteck *et al.*, 1985). Similarly an inverse correlation between tumour incidence and tissue histamine levels was reported for fibrosarcomas and Lewis lung carcinomas (Burtin *et al.*, 1985). The importance of

**Table I** Effect of rat mast cell products (MCP) on collagenase production by rat fibroblasts and tumour cells.

| | *Collagenase activity (Units mg$^{-1}$ protein)* |
|---|---|
| Fibroblasts (NRS) | |
| Control culture | 0.68 ± 0.12 |
| Culture + MCP | 5.36 ± 0.9[a] |
| Tumour Cells (MTLn3) | |
| Control culture | 0.36 ± 0.03 |
| Culture + MCP | 4.3 ± 0.5[a] |
| Control culture medium + MCP | < 0.06 |

Conditioned culture medium, with and without added MCP (2.5%, v/v), was assayed for total collagenase as described in **Methods.** Data represent the mean values of triplicate determinations ± s.e.
[a]$P < 0.002$; [b]$P < 0.001$ (Student's *t*-test).

**Table II** Activation of latent collagenase derived from rat fibroblasts (NRS) and tumour cells (MTLn3) by exposure to rat mast cell products (MCP).

| | *Collagenase activity (Units mg$^{-1}$ protein)* |
|---|---|
| Fibroblasts (NRS) | |
| Unactivated medium | 0.32 ± 0.05 |
| + MCP | 0.70 ± 0.01[a] |
| Tumour cells (MTLn3) | 0.12 ± 0.03 |
| + MCP | 0.73 ± 0.02[a] |
| MCP control + control | < 0.07 |

Latent collagenase preparations were assayed for collagenase activity before and after exposure to MCP (2.5% v/v) as described in **Methods.** Data represent mean values of triplicate determinations ± s.e.
[a]$P < 0.0005$ (Student's *t*-test).

mast cells in local homeostasis, inflammation and tumour surveillance is supported by many studies (Lewis & Austin, 1981; Parwaresch *et al.*, 1985), but in direct contrast to the reports of a protective role against tumours some investigators have stressed an association between mast cells and rapid tumour growth (Csaba *et al.*, 1961; Farnoush & McKenzie, 1983). Both heparin and histamine have been reported to have mitogenic properties (Roche, 1985; Norrby, 1985; Norrby & Enestrom, 1985) and Hartveit (1981) reported that mast cell degranulation in human breast carcinomas was associated with areas of infiltrative growth. Such diverse findings suggest that a unitary explanation for the functional significance of mast cells in tumour cell invasion is unlikely. However, the 13762NF rat mammary adenocarcinoma has several histological similarities to human breast carcinomas (Hartveit, 1981; Hartveit & Sandstad, 1982; Hartveit *et al.*, 1984) and the studies reported here may therefore be more relevant to the pathology of these neoplasms.

Mast cell degranulation in the rat, elicited by compound 48/80, was reported to induce local proliferation of fibroblasts and mesothelial cells with depletion of extracellular matrix as judged by ultrastructural studies (Norrby & Enestrom, 1984). Similarly in the present study at the later stages of tumour growth an increasing frequency of mast cell degranulation was associated with connective tissue lysis and, significantly, the latter was never observed around intact mast cells. The extent of degranulation was variable, not only between different tumour specimens but also at different locations in the same tumour. At present the stimulus for degranulation has not been identified, but the histological findings suggest a micro-environmental response at the tumour:host junction which possibly relate to local changes in homeostasis.

The degradation or lysis of connective tissue associated with mast cell degranulation may have several explanations. It is known that mast cells release a variety of proteolytic enzymes (Lagunoff, 1968; Birkedal-Hansen *et al.*, 1976; Keiser, 1980; Metcalfe & kaliner, 1981; Schwartz, 1983) and the tryptase of human mast cells, which is apparently resistant to plasma antiproteinases (Schwartz *et al.*, 1981), may contribute to fibrinolysis (Schwartz *et al.*, 1985), matrix destruction and the activation of cascade mechanisms. We have been unable to demonstrate detectable levels of a true collagenase in rat MCP, although the recent identification of human mast cell elastase (Meier *et al.*, 1985) could conceivably contribute collagenolytic activity. However, perhaps of greater relevance to *in vivo* collagenolysis is the finding that mast cell products significantly stimulated collagenase production by stromal fibroblast and tumour cell cultures, an observation previously reported for synovial fibroblasts (Yoffe *et al.*, 1984). As yet the stimulatory factor has not been identified, but the response of tumour cell lines to mast-cell mediated stimulation of collagenolysis appears to be related to the metastatic potential of the tumour cell (Dabbous *et al.*, 1986). The MCP preparations used in this study also proved effective activators for latent collagenases derived from fibroblast and tumour cells, presumably mediated via mast cell proteases as described previously (Birkedal-Hansen *et al.*, 1976). Thus the potential for MCP to stimulate latent collagenase production by surrounding fibroblasts or tumour cells, and subsequently to activate the released precursor, provides one effective mechanism for the generation of local collagenolytic activity. Another pathway is suggested by the report that macrophages are stimulated by MCP, probably heparin, to produce increased amounts of interleukin-1 (Yoffe *et al.*, 1985), a factor known to stimulate collagenase production by fibroblasts and tumour cells (Henry *et al.*, 1983).

Many studies have examined the role of collagenolytic enzymes in tumour invasion and metastatic spread (for reviews see Woolley *et al.*, 1980; Liotta *et al.*, 1982; Woolley, 1984) and recently several investigations have emphasised the importance of tumour:host cell interactions in connective tissue degradation (Tarin, 1976; Dabbous *et al.*, 1977, 1983*a, b*; Biswas, 1982; Henry *et al.*, 1983). The tumour:host interface or 'invasion zone' (Strauli, 1980) of many invasive tumours is variable with regard to the type and relative numbers of host cells. Previous immunolocalisation studies have demonstrated that collagenase production at the tumour edge is often microenvironmental in nature, but the cellular origin of the enzyme may depend to some extent on the type or tissue location of the invasive tumour (Woolley, 1982; Woolley & Grafton, 1980; Barsky *et al.*, 1983). Further enzyme localisation studies in conjunction with the identification of specific host cells should help to elucidate which cellular interactions are involved in generating local collagenolysis *in vivo*. However, at present our histological findings suggest that degradation of local collagenous matrix is a common feature of mast cell degranulation, an observation possibly related to the release of mast cell enzymes and/or the potential of this cell to modulate the expression of collagenolytic activity by surrounding cells.

This work was supported by USPHS National Cancer Institute grants RO1-CA25167 (Dabbous) and RO1-CA28844 (Nicolson).

### References

ATKINS, F.M., FRIEDMAN, M.M., SUBBA RAO, P.V. & METCALFE, D.D. (1985). Interactions between mast cells, fibroblasts and connective tissue components. *Inter. Arch. Allergy Appl. Immunol.*, **7**, 96.

BARSKY, S.H., TOGO, S., GARBISA, S. & LIOTTA, L.A. (1983). Type IV collagenase immunoreactivity in invasive breast carcinoma. *Lancet*, **i**, 296.

BEELEN, H.J. & WALKER, W.S. (1986). Isolation of rat peritoneal lymphocytes, eosinophilic granulocytes, mast cells and functionally distinct macrophage subpopulations by Percoll density gradients and centrifugal elutriation. *J. Immun. Meths*, (in press).

BIRKEDAL-HANSON, H., COBB, C.M., TAYLOR, R.E. & FULLMER, H.M. (1976). Activation of fibroblast procollagenase by mast cell proteases. *Biochim. Biophys. Acta.*, **438**, 273.

BISWAS, C. (1982). Tumor cell stimulation of collagenase production by fibroblasts. *Biochem. Biophys. Res. Commun.*, **109**, 1026.

BROMLEY, M., FISHER, W.D. & WOOLLEY, D. (1984). Mast cell at sites of cartilage erosion in the rheumatoid joint. *Ann. Rheumatoid Dis.*, **43**, 76.

BROMLEY, M. & WOOLLEY, D.E. (1984). Histopathology of the rheumatoid lesion: Identification of cell types at sites of cartilage erosion. *Arthritis Rheum.*, **27**, 857.

BURTIN, C., PONVERT, C., FRAY, A. & 5 others (1985). Inverse correlation between tumour incidence and tissue histamine levels in w/w$^{v}$, w$^{v}$/+ and +/+ mice. *J. Natl Cancer, Inst.*, **74**, 671.

CSABA, G., ACS, T., HORVATH, C. & MOLD, K. (1961). Genesis and function of mast cells: Mast cell and plasmacyte reaction to induced, homologous and heterogeneous tumors. *Br. J. Cancer*, **15**, 327.

DABBOUS, M.Kh., ROBERTS, A.N. & BRINKLEY, Sr.B. (1977). Collagenase and neutral protease activities in cultures of rabbit VX-2 carcinoma. *Cancer Res.*, **37**, 3537.

DABBOUS, M.Kh., EL-TORKY, M., HANEY, L., BRINKLY, Sr.B. & SOBHY, N. (1983a). Collagenase activity in rabbit carcinoma: Cell source and cell interactions. *Int. J. Cancer*, **31**, 357.

DABBOUS, M.Kh., EL-TORKY, M., HANEY, L., SOBHY, N. & BRINKLY, Sr.B. (1983b). Separation of VX-2 rabbit carcinoma-derived cells capable of releasing collagenase. *Exp. Mol. Path.*, **38**, 1.

DABBOUS, M.Kh., WOOLLEY, D.E., HANEY, L., CARTER, L.M. & NICOLSON, G.L. (1986). Host mediated effectors of tumor invasion: Role of mast cells in matrix degradation. *Clin. Exp. Metast.*, **4**, 141.

EHRLICH, P. (1879). Beitrage zur Kenntuiss der grannlierten Bindegewabzellen und der eosinophilen leucocyten. *Arch. Anat. Physiol.*, **1879**, 166.

FARNOUSH, A. & McKENZIE, I.C. (1983). Sequential histological changes and mast cell response in skin during chemically-induced carcinogenesis. *J. Path.*, **12**, 300.

FARNOUSH, A. & McKENZIE, I.C. (1984). Proliferation of mast cells in normal and DMBA-treated mouse skin. *J. Oral Pathol.*, **13**, 359.

FARRAM, E. & NELSON, D.S. (1980). Mouse mast cells as anti-tumor effector cells. *Cell. Immunol.*, **55**, 294.

FISHER, E.R. & FISHER, B. (1965). Role of mast cells in tumor growth. *Arch. Path.*, **79**, 185.

HARTVEIT, F. (1981). Mast cells and metachromasia in human breast cancer: Their occurance, significance and consequences: A preliminary report. *J. Path.*, **134**, 7.

HARTVEIT, F. & SANDSTAD, E. (1982). Stromal metachromasia: A marker for areas of infiltrating tumor growth? *Histopath.*, **6**, 423.

HARTVEIT, F., THORESEN, S., TANGEN, M. & MAARTMANN-MOE, H. (1984). Mast cell changes and tumour dissemination in human breast carcinoma. *Invasion Metastasis*, **4**, 146.

HENRY, N., VAN LAMSWEERDE, A.L. & VAES, G. (1983). Collagen degradation by metastatic variants of Lewis lung carcinoma: Co-operation between tumor cells and macrophages. *Cancer Res.*, **43**, 5321.

JANES, J. & MCDONALD, J.R. (1948). Mast cells: Their distribution in various human tissues. *Arch. Path.*, **45**, 622.

KEISER, H.D. (1980). The effects of lysosomal enzymes on extracellular substances. In *The Cell Biology of Inflammation*, Weissman G. (ed), p. 431. Elsevier/North Holland: Amsterdam.

LEWIS, R.A. & AUSTEN, K.F. (1981). Mediation of local homeostasis and inflammation by leukotrienes and other mast cell compounds. *Nature*, **293**, 103.

LIOTTA, L.A., THORGEIRSSON, U.P. & GARBISA, S. (1982). Role of collagenases in tumor cell invasion. *Cancer Metastasis Rev.*, **1**, 277.

MEIER, H.L., HECK, L.W., SCHULMAN, E.S. & MACGLASHAN, D.W. (1985). Purified human mast cells and basophils release human elastase and cathepsin G by an IgE-mediated mechanism. *Int. Arch. Allergy Appl. Immunol.*, **77**, 179.

METCALFE, D.D. & KALINER, M. (1981). Mast cells and basophils. In *Cellular Functions in Immunity and Inflammation*, Oppenheim, J.J. *et al.* (eds), p. 301. Edward Arnold: London.

NERI, A. & NICOLSON, G.L. (1981). Phenotypic drift of metastatic and cell surface properties of mammary adenocarcinoma cell clones during growth in vitro. *Int. J. Cancer.*, **28**, 731.

NERI, A., WELCH, D.R., KAWAGUCHI, T. & NICOLSON, G.L. (1982). Development and biologic properties of malignant cell sublines and clones of a spontaneously metastasizing rat mammary adenocarcinoma. *J. Natl Cancer Inst.*, **68**, 507.

NORRBY, K. (1985). Evidence of mast cell histamine being mitogenic in intact tissue. *Agents Actions*, **16**, 287.

NORRBY, K. & ENESTROM, S. (1984). Cellular and extracellular changes following mast-cell secretion in a vascular rat mesentary. *Cell Tissue Res.*, **235**, 339.

PARWARESCH, M.R., HORNBY, H.-P. & LENNART, K. (1985). Tissue mast cells in health and disease. *Path. Res. Pract.*, **179**, 439.

PILLARISETTI, V., SUBBA RAO, P.V., FRIEDMAN, M.M., ATKINS, F.M. & METCALFE, D.D. (1983). Phagocytosis of mast cell granules by cultured fibroblasts. *J. Immunol.*, **130**, 341.

ROCHE, W.R. (1985). Mast cells and tumors: The specific enhancement of tumor proliferation in vitro. *Am. J. Path.*, **119**, 57.

SCHITTECK, A., ABON ISSA, H., STAFFORD, J.H., YOUNG, D., ZWILLING, B. & JAMES, A.G. (1985). Growth of pulmonary metastases of B16 melanoma in mast cell-free mice. *J. Surg. Res.*, **38**, 24.

SCHWARTZ, L.B. (1983). Enzyme mediators of mast cells and basophils. *Clin. Rev. Allergy.*, **1**, 397.

SCHWARTZ, L.B., LEWIS, R.A. & AUSTEN, K.F. (1981). Tryptase from human pulmonary mast cells: purification and characterisation. *J. Biol. Chem.*, **256**, 11939.

SCHWARTZ, L.B., BRADFORD, T.R., LITTMAN, B.H. & WINTROUB, B.U. (1985). The fibrinolytic activity of purified tryptase from human lung mast cells. *J. Immunol.*, **135**, 2762.

SMYTH, C.J. & GUM, O.B. (1958). Mast cells in connective tissue diseases. *Arthritis Rheum.*, **1**, 178.

STRAULI, P. (1980). A concept of invasion. Proteases and Tumor Invasion. EORTC Monograph Series, Strauli, P. *et al.* (ed), vol. **6**. p. 1. Raven Press: New York.

SYLVEN, B. (1945). Ester sulphuric acids of high molecular weight and mast cells in mesenchymal tumors. *Acta Radiol.* (Suppl.), **59**, 93.

TARIN, D. (1976). Cellular interactions in neoplasia. In *Fundamental Aspects of Metastasis*, Weiss. L. (ed), p. 151. North Holland: Amsterdam.

WELCH, D.R., NERI, A. & NICOLSON, G.L. (1983). Comparison of 'Spontaneous' and 'Experimental' metastasis using Rat 13762 mammary adenocarcinoma metastatic cell clones, *Invasion Metastasis*, **3**, 65.

WOOLLEY, D.E. (1982). Collagenase immunolocalization studies of human tumours. In *Tumor Invasion and Metastasis*, Liotta L.A. & Hart I.R. (eds), p. 391. Martinus Nijhoff: The Hague.

WOOLLEY, D.E. (1984). Collagenolytic mechanisms in tumor cell invasion. *Cancer Metastasis Rev.*, **3**, 61.

WOOLLEY, D.E. & GRAFTON, C.A. (1980). Collagenase immunolocalisation studies of cutaneous secondary melanomas. *Br. J. Cancer*, **42**, 260.

WOOLLEY, D.E., TETLOW, L.C., EVANSON, J.M. (1980). Collagenase immunolocalisation studies of rheumatoid and malignant tissues. In *Collagenase in Normal and Pathological Connective Tissues*, Woolley, D.E. & Evanson, J.M. (eds), p. 105. Wiley: Chichester, UK.

WOOLLEY, D.E., TAYLOR, D.J. & YOFFE, J.R. (1984). Mammalian collagenases; aspects of regulation. *Life Chem. Rep.*, **2**, 181.

WESTPHAL, E. (1891). Uber mastzellen: In *Farbenanalytische Untersuchuugen*, Hirschwald, E.P. (ed), p. 17. Berlin.

YOFFE, J.R., TAYLOR, D.J. & WOOLLEY, D.E. (1984). Mast cell products stimulate collagenase and prostaglandin E production by cultures of adherent rheumatoid synovial cells. *Biochem. Biophys. Res. Commun.*, **122**, 270.

YOFFE, J.R., TAYLOR, D.J. & WOOLLEY, D.E. (1985). Mast-cell products and heparin stimulate the production of mononuclear-cell factor by cultured human onocyte/macrophages. *Biochem. J.*, **230**, 83.